# Autonomous agents: Augmenting visual information with raw audio data

**DOI:** 10.1371/journal.pone.0318372

**Published:** 2025-05-23

**Authors:** Enoch Solomon

**Affiliations:** Department of Computer Science, Virginia State University, Petersburg, Virginia, United States of America; University of Foggia: Universita degli Studi di Foggia, ITALY

## Abstract

In the realm of game playing, deep reinforcement learning predominantly relies on visual input to map states to actions. The visual data extracted from the game environment serves as the primary foundation for state representation in reinforcement learning agents. However, humans leverage additional sensory inputs, such as audio cues, which play a pivotal role in perception and decision-making. Therefore, incorporating raw audio along with visual information shows potential for offering valuable insights to reinforcement learning agents. This study advocates for the integration of raw audio samples as complementary information to visual data in state representation. By using raw audio with visual cues, our objective is to enrich the decision-making process of the agent at each stage. Experimental evaluation were conducted employing Deep Q Networks (DQN) and Proximal Policy Optimization (PPO) algorithms within ViZDoom and Unity reinforcement learning environments. The results of our experiments reveal that augmenting visual information with raw audio samples yields superior rewards and expedites the learning rate compared to relying solely on visual data. Additionally, the findings suggest that considering both visual and audio features enhances the agent’s behavior, a trend observed across Unity and ViZDoom environments. This study underscores the potential advantages of incorporating multisensory information, particularly raw audio, into the state representation of reinforcement learning agents. Such insights contribute to advancing our understanding of how agents perceive and engage with their environments, ultimately enhancing performance in complex gaming scenarios.

## 1 Introduction

Reinforcement learning (RL) constitutes a key domain within machine learning, where an agent learns through iterative interactions with an environment to accomplish specific objectives. It delves into the study of problems and algorithms geared towards shaping policies that optimize decision-making to maximize rewards obtained from the environment [1–3]. Central to RL is the conceptual framework of a Markov decision process, which models the problem to be resolved [4,5]. Core components encompass agents, environments, states, actions, and rewards, orchestrating a sequence where an agent selects actions, transitions to new states, and receives rewards contingent on its decisions.

Successful applications of RL span various domains, ranging from solving physics-based control problems [6] to diverse applications like Atari games [7], robotic manipulation [8], self-driving vehicles [9], and intelligent agents for 2D, 3D, and virtual reality games [10,11]. The crux of RL lies in formulating policies that optimize long-term rewards from the environment [2,3]. An optimal policy guides the agent to navigate the environment efficiently, maximizing cumulative rewards across episodes by considering future scenarios and rewards rather than solely focusing on immediate gains.

Recent advances in deep learning have unlocked the ability to extract high-level features from raw sensory data, propelling advancements in computer vision [12–17] and speech recognition [18]. In particular, convolutional neural networks (CNNs) have demonstrated remarkable prowess in visual recognition tasks [19] and audio analysis [20,21]. Despite these breakthroughs, existing RL approaches predominantly rely on visual information for state representation [22], overlooking the potential benefits of integrating audio cues.

However, human perception benefits from diverse sensory inputs, including auditory cues, which are pivotal in providing crucial gameplay cues and environmental awareness in gaming contexts [23]. Audio cues, such as sounds and music, are instrumental in conveying environmental information to agents [24]. For example, audio cues can signal changes in direction, alerting the agent to potential dangers or opportunities. The absence of such auditory inputs in RL models utilizing only visual information may hinder optimal performance and impede learning efficiency, particularly in scenarios where critical events may be missed, as in the case of a loud crash occurring out of sight. Moreover, the incorporation of audio cues is indispensable for enhancing gaming experiences for visually impaired individuals [25].

Reinforcement Learning (RL) has been applied in various multi-modal learning tasks. For example, [26] utilized RL to improve machine translation across different data modalities. Similarly, [27] applied RL to image-based question answering, an inherently multi-modal learning task. RL has also been used in the context of visual dialogues, integrating multiple modalities [28]. However, none of these studies have explored the use of RL for Active Learning (AL) with multimodal data. The most similar approach to ours is the multi-view AL framework [29], which employs standard heuristic AL strategies to select data from multiple views.

Learning solely from raw visual data presents challenges, particularly in scenarios requiring intricate spatial navigation where direct line-of-sight is obstructed. For instance, consider a searching task where an agent equipped solely with visual information struggles to systematically locate a target. In such instances, leveraging raw audio samples alongside visual inputs could furnish valuable information, enhancing the agent’s decision-making capabilities. Accordingly, this work advocates for the integration of raw audio samples as supplementary input alongside visual information in RL tasks, exploring their impact on decision-making processes. Additionally, we evaluate the efficacy of using solely raw audio samples in gaming environments.

Inspired by the natural inclination to guide others using spoken language, prompting the query, "Why do autonomous agents not leverage auditory cues?", our investigation aims to shed light on the untapped potential of incorporating auditory inputs directly into RL frameworks.

To demonstrate the efficacy of integrating raw audio samples into reinforcement learning tasks, we assess the proposed system’s performance across two prominent reinforcement learning environments: ViZDoom [30] and Unity ML [10,31]. Our experiments encompass two main setups: utilizing only visual information and incorporating both visual data and audio features. The experimental outcomes, conducted within both Unity and ViZDoom environments, reveal that a reinforcement learning agent trained using visual information alongside raw audio samples achieves superior average rewards and demonstrates accelerated learning compared to relying solely on visual inputs.

This work makes significant strides in the field of reinforcement learning (RL) by addressing the limitations of conventional approaches that predominantly rely on visual information for state representation. While deep reinforcement learning has shown remarkable success in various domains, its reliance on visual data alone overlooks the potential benefits of incorporating additional sensory inputs, such as audio cues. Human perception and decision-making are inherently multisensory, integrating auditory, visual, and other sensory information to navigate complex environments effectively. Inspired by this natural multisensory integration, our research proposes and validates a novel framework that combines raw audio samples with visual data, aiming to enhance the performance and learning efficiency of RL agents.

The main contributions of this work are multi-faceted:

- **Innovative Multisensory Integration Framework:** We introduce and validate a framework that combines raw audio cues with visual information to enrich the state representation of RL agents. This approach leverages the complementary nature of auditory and visual inputs, reflecting the multifaceted way humans perceive and interact with their environments.
- **Comprehensive Experimental Evaluation:** Using Deep Q Networks (DQN) and Proximal Policy Optimization (PPO) algorithms, we rigorously evaluate the proposed multisensory framework in two prominent RL environments: ViZDoom and Unity ML. Our experimental results demonstrate that incorporating raw audio samples alongside visual data leads to significantly higher average rewards and accelerates the learning process compared to agents relying solely on visual inputs. This empirical validation highlights the practical benefits of multisensory integration in complex RL tasks.
- **Enhanced Agent Performance and Behavior:** Our findings reveal that the addition of audio features substantially improves agent performance. Specifically, agents trained with both visual and audio inputs exhibit more effective decision-making and behavior, overcoming limitations of visual-only models, such as missing critical events or navigating challenging scenarios where visual information is obstructed.
- **Advancement of Sensory Integration in RL:** This study underscores the critical importance of incorporating diverse sensory inputs into RL frameworks. By demonstrating the advantages of raw audio in state representation, we provide new insights into how RL agents can better perceive and engage with their environments. This advancement is particularly relevant for scenarios where visual data alone is insufficient, such as in environments with obstructed views or for individuals with visual impairments.
- **Future Research Directions:** Our work opens several avenues for future exploration. These include investigating the specific types of auditory cues that are most beneficial for decision-making, evaluating the impact of audio on various neural network architectures, such as recurrent neural networks, and extending our approach to other RL tasks and environments. Additionally, comparing agents with and without audio inputs using advanced neural network models could provide deeper insights into how multisensory data influences learning dynamics.

In summary, this work makes a significant contribution by demonstrating the value of integrating raw audio with visual information in reinforcement learning. Our findings advance the field by enhancing agent performance and providing a foundation for future research into multisensory integration in complex RL environments. This work not only enhances current RL methodologies but also opens up exciting new avenues for research and application, ultimately contributing to the development of more sophisticated and capable AI systems.

The remainder of this paper is as follows. The subsequent sections provide an overview of the deep reinforcement learning techniques employed in our experiments and delineate the architecture of the proposed multimodal reinforcement learning system. Sections V and VI discuss into the experimental results and draw conclusions, respectively.

## 2 Deep reinforcement learning

Reinforcement learning methods are designed to tackle tasks within a given environment by accumulating experiences from interactions with that environment and subsequently learning from them [2,32]. The environment is typically formalized as a Markov Decision Process (MDP), characterized by a set of possible states s∈S, a set of possible actions a∈A, a reward function $R(s_{t},a_{t},t_{t+1})\in \mathbb{R}$, and a transition distribution between states $P(s_{t+1}|s_{t},a_{t})\in [0,1]$. The notation distinguishes values at different time steps, denoted by t∈ℕ. Within each state, a policy π:s→a dictates the selection of an action *a*_*t*_, following which the environment transitions to the next state $s_{t+1}\sim P(s_{t+1}|s_{t},a_{t})$. Subsequently, the policy (agent) receives a reward $r_{t}=R(s_{t},a_{t},s_{t+1})$ based on this experience. To streamline the discussion, we focus on *episodic games*, wherein the game concludes upon the agent reaching a terminal state.

MDP within reinforcement learning enhances the agent’s ability to make decisions based on richer, multi-modal sensory inputs. The process involves combining the strengths of both types of data, requiring careful design of neural architectures, efficient learning algorithms, and methods to handle temporal and spatial dependencies across modalities. By addressing these challenges, agents can perform more effectively in real-world environments, where multi-modal data is often essential for accurate perception and decision-making.

The goal of reinforcement learning is to learn a policy that maximizes the return from any time step t∈ℕ until the end of the episode T∈ℕ

$$G_{t}=\sum_{k=0}^{T}\gamma^{k}r_{t+k},$$ (1)

where the *discount factor* γ∈(0,1] is used to stabilize the learning and/or weight the importance of future states vs. nearby states.

*Proximal Policy Optimization* (PPO) [33] stands out as one of the most successful deep reinforcement learning methods, consistently achieving state-of-the-art performance across a diverse array of challenging tasks [33,34]. Notably, it has become one of the most widely adopted reinforcement learning algorithms within popular frameworks [10,35,36]. PPO combines elements from actor-critic and policy-gradient methods, leveraging a compact set of experiences from the environment before performing gradient ascent to maximize expected return (see Equation (1)). Moreover, PPO incorporates constraints to prevent drastic updates, a feature that has demonstrated enhanced results compared to standard actor-critic methods [33].

Reinforcement learning algorithms have proven successful in various domains, as evidenced by their application in tasks such as object localization under diverse surface conditions [37]. In this context, we employ the Actor-Critic approach for our PPO agent, which comprises two deep neural networks: the Actor and the Critic. The Actor learns to select actions based on observed environmental states, while the Critic evaluates the efficacy of these actions in improving the environment’s state. Interaction with the game environment occurs over a fixed number of steps, during which experiences are collected to update the models’ policies. In our setup, the reinforcement learning agent receives either RGB images of the game or raw audio samples as input and outputs one of the feasible actions.

Subsequently, the predicted action is executed in the game environment, and the resulting outcomes are observed. Positive outcomes yield rewards, which are then processed by the Critic model. The Critic’s primary function is to assess whether the action improves the environment’s state and provide feedback to the Actor accordingly. It generates a real-valued rating (Q-value) for the action taken in the preceding state. By comparing this rating, the Actor can refine its policy to make better decisions.

*Deep Q-networks* (DQN), introduced by [7], have enabled the application of Q-learning [39,42] in complex environments like video games. DQN is a model-free off-policy algorithm designed to estimate the long-term expected returns from executing actions in specific states. These estimated returns, known as Q-values, indicate the potential long-term rewards associated with particular actions. DQN leverages deep learning to estimate the value function instead of a discrete table, employs two techniques—replay memory and target networks—to stabilize learning. Notably, DQN has demonstrated success in playing various Atari games [7] and Doom [30]. Due to its efficacy and simplicity, we employ DQN in our experiments.

Integrating audio and visual information into Q-learning offers a more comprehensive understanding of the environment, which can significantly improve decision-making in tasks involving multi-modal sensory data. By combining convolutional and recurrent networks to process visual and auditory inputs, respectively, and updating Q-values based on the enriched state representation, agents can perform more robustly in real-world tasks where multi-modal cues are essential.

Q-learning [39] addresses this challenge by learning the *state-action value function* Q:s×a→G, which represents the expected return from taking action *a*_*t*_ in state *s*_*t*_. If the true state-action values were known, selecting the action with the highest value, $a_{t}=\mathrm{\backslash arg}\max_{a}Q(s_{t},a)$, would yield the optimal policy [39]. Q-learning achieves this by updating the value of each state-action pair iteratively based on experiences collected during interactions with the environment.

$$Q(s_{t},a_{t})=Q(s_{t},a_{t})+\alpha \left(r_{t}+\gamma \max_{a}Q(s_{t+1},a)-Q(s_{t},a_{t})\right)$$ (2)

where *the learning rate* α∈ℝ controls the learning rate. DQN extends this by using a deep neural network rather than a table to estimate the *Q*-function. This allows the use of Q-learning with high-dimensional states and actions (e.g. video games, where states are pixels of an image). The values of Q-values are learned iteratively by updating the current Q-value estimate towards the observed reward and the max Q-value over all actions a in state s. The update rule of DQN (Eq 2) is modified for training neural networks.

The Deep Q Network (DQN) relies on three key techniques to stabilize learning: Replay Memory: Experiences are stored in a replay memory and sampled uniformly during training. This allows the agent to learn from a diverse set of past experiences, mitigating the impact of temporal correlations in sequential data. Target Network: A separate target network is employed to provide updates to the main network. This helps stabilize training by decoupling the target Q-values from the current parameters of the main network, reducing the risk of divergence during training. Adaptive Learning Rate: An adaptive learning rate method, such as RMSProp [43], maintains a per-parameter learning rate and adjusts the learning rate according to the history of gradient updates to that parameter. This adaptive scheme improves the efficiency of learning by dynamically adjusting the learning rates based on the local geometry of the loss landscape.

To encourage exploration during both training and testing, we employ an ϵ-greedy exploration strategy. The DQN selects a random action with probability ϵ, and the optimal action with probability 1−ϵ. Initially, ϵ is set high at the beginning of training to encourage exploration, and then annealed gradually towards zero over training to shift towards exploitation, favoring optimal actions. During testing, the value of ϵ is fixed to a low value, typically 0.05, to prioritize exploitation and ensures consistent performance.

In this work, we utilize DQN and Proximal Policy Optimization (PPO) agents as the basis for experiments in ViZDoom and Unity ML environments, respectively. These algorithms are chosen for their versatility in handling different types of data, including visual information and raw audio samples.

## 3 Proposed multimodal reinforcement learning system architecture

Multimodal Reinforcement Learning (MRL) involves leveraging multiple input modalities—such as images, audio, and text—to improve the performance of reinforcement learning (RL) agents. In environments where both visual and auditory information is crucial (e.g., autonomous driving with sound-based navigation cues or interactive robotics using both vision and sound), integrating these different types of sensory inputs can lead to more robust and efficient learning.

In this proposed method, we will explore how Deep Reinforcement Learning (DRL) can be enhanced by integrating both image (vision) and audio (sound) inputs. This involves creating a multimodal representation that fuses visual and auditory data, enabling the agent to make decisions based on a richer understanding of its environment.

This work introduces the utilization of raw audio samples in reinforcement learning tasks, implemented in both ViZDoom and Unity ML environments. In both environments, raw audio samples are integrated alongside visual information. Notably, the impact of solely utilizing raw audio samples is evaluated specifically in the Unity environment.

**Fig 1:**
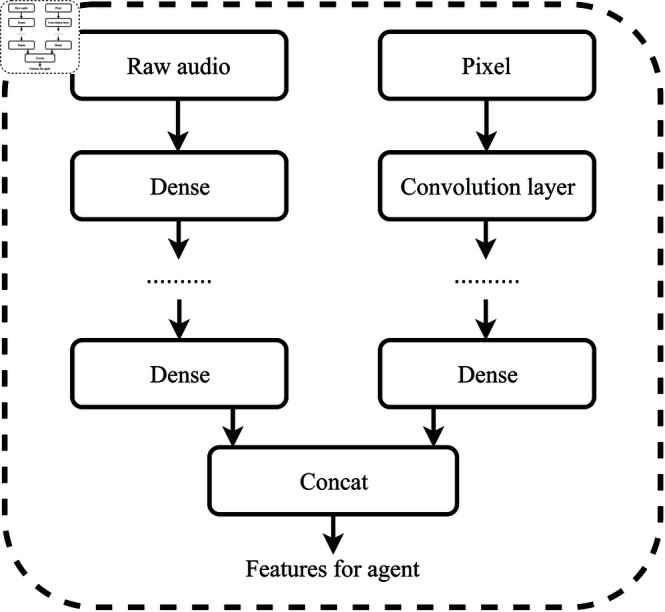
Proposed reinforcement learning architecture.

Fig 1 illustrates the architecture of the proposed reinforcement learning system, which incorporates two input sources: pixels and raw audio samples. These sources of learned features are combined in the final layer of the network by concatenating the outputs of two dense networks. The system leverages both Deep Q Network (DQN) and Proximal Policy Optimization (PPO) algorithms to validate its performance.

The proposed method integrates convolutional neural networks (CNNs) for processing image data and LSTMs (Long Short-Term Memory), for processing audio data. The fused features from both modalities are used in the reinforcement learning model. The image is passed through several convolutional layers followed by pooling layers, ending in a fully connected layer to extract high-level features. Whereas the audio input is converted into a spectrogram (a 2D representation of the frequency content of audio over time). This representation transforms raw audio into a form that a neural network can process more easily, LSTMs, can be used to capture temporal dependencies in the audio signals, allowing the network to understand sequences of auditory events. After extracting the features from both the image and audio, the next step is to combine them into a unified representation for the RL agent to make decisions.

$$Fusedrepresentation:𝐳=\left[{𝐳}_{\text{image }},{𝐳}_{\text{audio }}\right]$$ (3)

where ${𝐳}_{\text{image }}$ is the feature vector from the CNN and ${𝐳}_{\text{audio }}$ is the feature vector from the LSTM.

In the ViZDoom environment [30], where direct access to raw audio sources is not feasible, we simulate audio by calculating the distance between the agent and the target (i.e., distance to the goal) and incorporating these values into the neural network. This approach allows us to incorporate audio-like features into the learning process, with the magnitude of the samples increasing as the agent approaches the goal.

The loss function for training the RL models for DQN and PPO are described below respectively.

$$L_{DQN}=𝔼\left[{\left(Q_{\text{current }}\left(s_{t},a_{t}\right)-\left(r_{t}+\gamma \max_{a^{\prime }}Q_{\text{target }}\left(s_{t+1},a^{\prime }\right)\right)\right)}^{2}\right]$$ (4)

$$L_{PPO}(\theta )={𝔼}_{t}\left[\min\left(\frac{\pi_{\theta }\left(a_{t}|s_{t}\right)}{\pi_{\theta_{0ld}}\left(a_{t}|s_{t}\right)}A_{t},\mathrm{clip}\left(\frac{\pi_{\theta }\left(a_{t}|s_{t}\right)}{\pi_{\theta_{old}}\left(a_{t}|s_{t}\right)},1-\epsilon ,1+\epsilon \right)A_{t}\right)\right]$$ (5)

Hence, since Unity environment [10] provides direct access to audio generated by the Unity engine, enabling us to seamlessly integrate raw audio samples. Here, the audio source is attached to the goal object, while an audio listener is attached to the agent. As the agent navigates the environment, the volume of the audio clip dynamically adjusts based on the agent’s proximity to the goal object. The raw audio samples extracted from the audio clip are fed into the neural network alongside the raw visual information at each step of the learning process. These raw audio features, representing the signal itself, are appropriately normalized to [0,1] for neural network processing. By directly utilizing raw audio samples, the need for feature extraction is circumvented, although this approach may present challenges due to the complexity of the data for the learning algorithm.

## 4 Experimental setup

In this section, we outline the experimental setups for both the ViZDoom and Unity environments. ViZDoom is chosen for its versatility, ease of use, and efficient 3D platform, allowing for the development of artificial intelligence bots that navigate Doom [38] using only visual information (i.e., the screen buffer). This environment is particularly well-suited for research in vision-based machine learning and deep reinforcement learning.

Both the Unity and ViZDoom agents are trained using LSTMs (Long Short-Term Memory) recurrent neural networks for processing audio samples and CNN architectures for handling visual information. The agent relies on observations of its environment to infer the state of the world. In Unity ML, observations can be visual or vector-based, while ViZDoom exclusively utilizes visual observations, with the camera image attached to the agent.

The experiments were conducted on a machine equipped with a 16-core Intel Xeon processor, Nvidia RTX2080ti GPU, CUDA 12.3, Python 3.6, TensorFlow 2.13, and Ubuntu 18.04.

To enhance learning stability, the replay technique is applied to store the agent’s experiences and randomly select batches for network training in the DQN implementation. This technique prevents the network from solely focusing on its immediate actions in the environment, enabling it to learn from a broader range of past experiences. Each experience is stored as a tuple of <*state*, *action*, *reward*, *next state*>. The experience replay buffer maintains a fixed number of recent memories, replacing old ones as new experiences are added. This approach ensures that the network learns from a diverse set of past interactions, contributing to more robust learning.

### 4.1 Scenarios

We have used two different scenarios for the ViZDoom and Unity ML environment.

#### 4.1.1 ViZDoom.

One of the most important features of ViZDoom is its ability to run custom scenarios. This ViZDoom offers the capability to run custom scenarios, enabling the creation of tailored maps, environment mechanics, terminal conditions, and rewards. We leveraged this feature by creating our own scenario map using SLADE.

Our custom scenario map was designed to evaluate the performance of our proposed approach. In this scenario, the agent’s objective is to navigate through five rooms and locate the target. The episode terminates either when the agent reaches the goal or upon reaching a timeout. Upon successfully reaching the goal, the agent receives a reward of 1. However, a penalty of –1 is incurred on every game tick.

#### 4.1.2 Unity.

**Fig 2:**
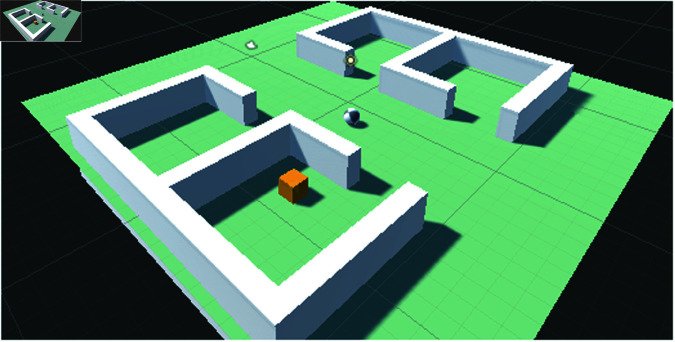
The scenario used in Unity environment. Both the agent and target are spawned randomly at the start of the game anywhere in the game environment for each episode.

**Fig 3:**
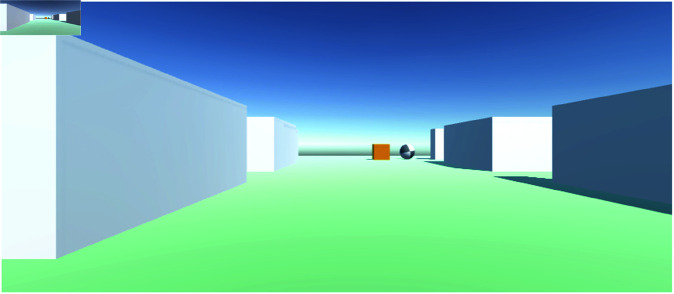
The images shown correspond the downscaled images an agent receives in Unity environment.

Similarly, we developed our own scenario in Unity ML (depicted in Figs 2 and 3) comprising four small rooms. At the start of the game, both the agent and the target are randomly spawned in any of the four rooms or outside. The agent must navigate through the rooms to locate the target. This environment is characterized by sparse rewarding, as the agent only receives a positive reward upon reaching the goal. The episode concludes upon the agent reaching the goal or upon reaching a timeout. Upon successfully reaching the goal, the agent receives a reward of 1, otherwise, it receives 0. Notably, when raw audio samples are utilized in the Unity ML environment, the audio source is attached to the target, while the audio listener is attached to the agent.

In our study, we chose different experimental settings to accommodate the unique requirements and characteristics of the algorithms employed in the ViZDoom and Unity ML environments. ViZDoom utilizes a modified version of the Deep Q-Network (DQN) algorithm, which is well-suited for handling the high-dimensional visual inputs of first-person shooter games. Conversely, the Unity ML environment allows for the implementation of Proximal Policy Optimization (PPO), a policy gradient method known for its stability and efficiency in continuous action spaces. By selecting these algorithms, we ensure that each environment is optimally addressed, leveraging the strengths of DQN in discrete action settings and PPO in more complex, multi-agent scenarios. Additionally, both algorithms utilize tailored neural network architectures that are specifically designed to process the multimodal inputs—raw audio and visual data—maximizing the agents’ learning capabilities and performance in their respective environments. This careful consideration of experimental settings and neural network choice underscores our commitment to achieving robust and generalizable results across diverse reinforcement learning tasks.

### 4.2 Training

ML-Agents [10] offer an implementation of the Proximal Policy Optimization (PPO) reinforcement learning algorithm, utilizing a neural network to approximate the optimal function mapping an agent’s observations to the most advantageous action given a state. This PPO implementation within ML-Agents is built using TensorFlow and operates within a separate Python process.

The training process begins with the initialization of parameters such as the discount factor and learning rate. Training proceeds over multiple epochs, each comprising numerous learning steps (e.g., 500,000 training steps). Initially, the agent takes random actions to explore the environment, followed by actions selected based on the learned policy to exploit the environment’s rewards. At the conclusion of each training epoch, the network weights are saved, and these saved models are subsequently evaluated to assess the final performance of the trained agent.

In both the Unity and ViZDoom environments, Convolutional Neural Network (CNN) architectures are employed for processing visual information, while fully connected layers are utilized for handling raw audio samples. When raw audio samples are combined with visual information, each stream is initially processed independently before being merged at the final layer, as illustrated in Fig 1. The first convolutional layer for both images and raw audio samples comprises 16 filters of size 8 and a stride of 4, followed by a second convolutional layer with 32 filters of size 4 and a stride of 2. Subsequently, a fully connected layer transforms the input to 512 units, which are further processed by another fully connected layer to yield an output size equal to the number of actions available in the game.

### 4.3 Hyperparameters

The hyperparametes of Unity and ViZDoom are described as follows:

#### 4.3.1 Unity.

We identified the hyperparameters that most affect the model’s performance in the Unity ML environment such as batch size, beta, buffer size, epsilon, lambda, learning rate, number of epochs, time horizon (number of steps of experience to collect per agent before adding it to the experience buffer) and sequence length. For each hyperparameter, we defined a reasonable search space (range of values). Then, we leverage the gradient-based optimization search strategy. We used cross-validation to evaluate the model’s performance for each hyperparameter configuration. This helps in assessing the generalization ability of the model and reduces overfitting to a specific validation set. For each combination of hyperparameters, we train the model on the training data and evaluate it on the validation data. Then we compare the performance of different hyperparameter configurations and chose the configuration that yields the highest performance. Once we identified the best hyperparameters, we retrain the model using the full training dataset and evaluate its performance on a separate test set to check for generalization.

ML-Agents also offer modularity in defining reward signals. In this work, we utilize two reward signals: extrinsic reward and curiosity [40], aiming to shape the agent’s behavior effectively. For the extrinsic reward, we set the strength to 1 and the discount factor to 0.99. Regarding the curiosity reward signal, the strength is set to 0.02, and the discount factor is 0.99. Additionally, we conduct an experiment without incorporating curiosity.

#### 4.3.2 ViZDoom.

For the ViZDoom experiment, we train the DQN agent using the RMSProp algorithm with minibatches of size 64. We store the most recent 10,000 timesteps in the replay memory. The discount factor is set to 0.99, while the learning rate remains fixed at 0.00025. During training, we employ an ϵ-greedy policy, where the agent selects a random action with probability ϵ. Initially, ϵ is set to 1.0 and gradually annealed to 0.1 after a certain number of steps.

In the ViZDoom experiments, we utilize a frame-skip of 10 frames, meaning the agent has the opportunity to select an action only every 10th game frame, corresponding to 285ms of in-game time, as the game runs at 35 frames-per-second. The action chosen in the previous step is repeated across all frames between these agent steps. Additionally, we adjust the magnitude of the audio based on the Euclidean distance from the agent to the goal. As ViZDoom does not allow direct inclusion of actual audio, we manipulate the vector values of raw audio accordingly. When the agent is close to the goal, the raw audio samples have higher values, whereas they have lower values when the agent is far.

Given the relatively high resolution image provided by the ViZDoom environment (640 x 480), we downsample the resolution to 80 x 60 at each frame step to reduce computational time during neural network training. Conversely, in the Unity game environment, we utilize an image size of 84 x 84 at each frame step, along with all three channels of the image. For the audio features, we feed vectors of size 100 raw audio samples at each time step.

### 4.4 Performance metric

To evaluate the performance of reinforcement learning, it’s common to plot the time versus episodic reward and observe how the agent’s performance evolves during training. In this work, we assess the average reward per episode to compare the performance of the baseline (without audio samples) and the proposed method. Since the average rewards of individual training runs can vary significantly, it’s typical to conduct multiple runs and average the results [41]. Therefore, the reported results in the following subsections are averaged over three experiment runs. In other words, each point in the learning curve figures represents the average of three episodes.

## 5 Experimental results

To assess the performance of the proposed approach, we initially trained a neural network model using only visual information in both ViZDoom and Unity ML environments. These experiments with image pixels serve as the baseline system. Subsequently, we compared the performance of incorporating raw audio samples alongside visual information in both ViZDoom and Unity ML environments. Additionally, we evaluated the impact of using raw audio samples alone in this study. For this evaluation, we employed both PPO and DQN algorithms to assess the performance of the proposed system across Unity and ViZDoom reinforcement learning environments.

**Fig 4:**
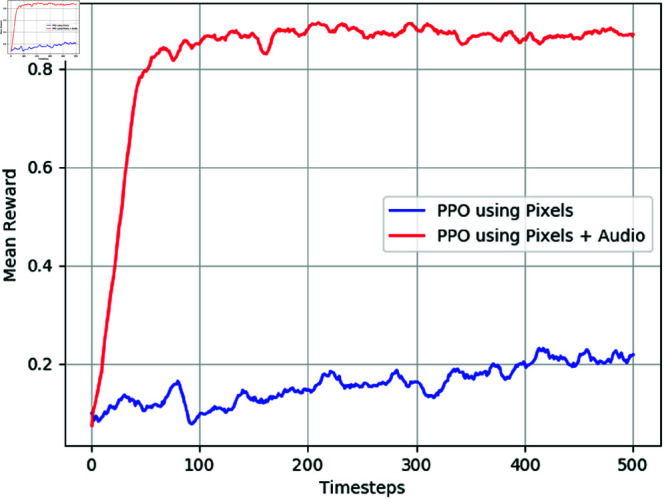
The mean reward results of using visual information and audio samples in the ViZDoom environment.

Fig 4 presents the experimental results of using visual information and raw audio samples in the ViZDoom environment with the PPO algorithm. The baseline system relies solely on visual information. The figure illustrates that the agent struggles to reach the target consistently when using only visual information. However, the addition of complementary raw audio samples alongside visual information enables the agent to reach the goal more reliably. Specifically, while using visual information and audio features provides an average reward of –550 during the last episodes, using only visual information yields an average reward of –1300 in the same episodes. Thus, the learning curves in Fig 4 demonstrate that leveraging visual information along with audio features results in better mean reward and a faster learning rate compared to using only visual information.

Furthermore, we conducted additional test experiments to evaluate the performance of the trained system. The test experiment involved running the trained model for 100 episodes. Table 1 displays the test results, comparing the average success rate of using visual information alone versus using visual information with raw audio samples. The results indicate that augmenting visual information with raw audio samples leads to a higher average success rate compared to using only visual information. Specifically, using only visual information results in an average success rate of 43%, whereas augmenting visual information with raw audio samples increases the average success rate to 87%. These results highlight the challenge faced by the agent in making intelligent moves to reach the target using only visual information in difficult scenarios.

**Table 1:** Average success rate (%) of the test experiment on ViZDoom and Unity environments.

gray Reinforcement Learning System	PPO with curiosity	PPO without curiosity	DQN
visual information	20	18	44
visual information + raw audio samples (25%)	92	94	86
visual information + raw audio samples (50%)	93	92	86
visual information + raw audio samples (75%)	95	96	88
visual information + raw audio samples (100%)	99	100	90

In addition to the ViZDoom environment, we also evaluated the impact of using raw audio samples for a searching task in the stable baselines environment. Consistent with the results observed in the ViZDoom environment, incorporating raw audio samples alongside visual information facilitated faster target acquisition compared to using only visual information, regardless of whether DQN or PPO learning algorithms were employed.

In the Unity ML experiments, we explored the use of curiosity reward signals to encourage exploration in environments with sparse extrinsic rewards. Consequently, we evaluated experiments both with and without curiosity reward signals and compared their performances, considering both visual information and raw audio samples.

**Fig 5:**
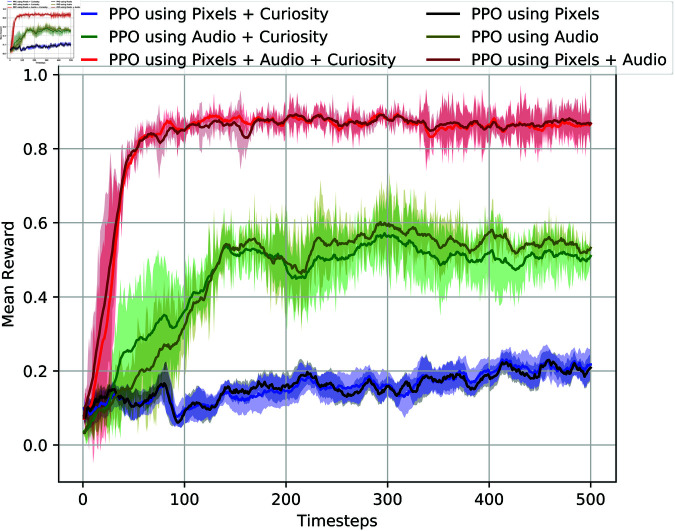
The mean and standard deviation of the test results are presented for the use of both visual information and raw audio samples in the Unity ML environment. The x-axis represents the timesteps, scaled by a factor of 100.

Fig 5 illustrates the experimental results of using visual information and audio samples in the Unity environment, both with and without curiosity. The baseline system relies solely on visual information. The figure indicates that the agent achieves better rewards when leveraging both raw audio samples and visual information, compared to using only pixel images, regardless of the presence of curiosity.

Specifically, for the PPO algorithm, using only visual information fails to reliably guide the agent to the target most of the time, as depicted in the figure. However, by incorporating raw audio samples alongside visual information, the agent consistently reaches the target, achieving near-maximum rewards. Notably, the learning curves show that while using only visual information requires 500,000 steps to achieve even a reward of 0.2, the combined use of visual and auditory information reaches maximum rewards within 100,000 steps.

Furthermore, the figure demonstrates that the average rewards obtained with and without the curiosity reward signals yield similar results in our experimental setup. Hence, the addition of raw audio samples to visual information significantly improves the agent’s performance in reaching the target, regardless of whether curiosity is employed or not.

The test experiment is carried out on 100 game episodes using the last training model. Raw audio samples (%) is the probability that the raw audio samples are used in the game environment. The visual information is used all the time (100%). The baseline system is the one based on only visual information.

The test results of 100 episodes using visual information, along with visual information with raw audio samples, with and without curiosity, are summarized in Table 1. The table clearly illustrates the stark difference in performance between the different setups.

When relying solely on visual information, the success rate is a mere 15%. However, integrating raw audio samples with visual information significantly improves the success rate to 100%, regardless of the presence of curiosity. This indicates that the addition of raw audio samples enables the agent to consistently reach the target, overcoming the limitations of relying solely on visual observations.

The results further reinforce the efficacy of incorporating raw audio samples alongside visual information for enhancing the agent’s performance in target-reaching tasks. Whether curiosity is employed or not, the addition of raw audio samples consistently enables the agent to successfully navigate the environment and reach the target in every episode, highlighting the robustness and effectiveness of the proposed approach.

The experimental results presented in Fig 5 reveal another intriguing finding: the use of only raw audio samples, both with and without curiosity, yields better average rewards compared to using only visual information. While the average reward peaks at 0.2 when relying solely on visual information, it significantly improves to 0.5 and 0.48 during the same training steps when using only audio samples, for setups with and without curiosity, respectively.

This observation suggests that audio samples play a crucial role in enhancing the agent’s performance, particularly in scenarios where visual information alone may be insufficient. Notably, when the target is spawned behind walls within rooms, the agent may struggle to navigate solely based on visual cues. However, the inclusion of audio samples provides additional environmental context, enabling the agent to make more informed decisions and achieve better rewards.

Furthermore, we compared the average total rewards of the last 100 episodes for both PPO (with and without curiosity) and DQN in both Unity ML and ViZDoom environments. Table 1 highlights that utilizing both visual information and raw audio samples consistently leads to better rewards compared to relying solely on visual information. This finding underscores the effectiveness of incorporating audio information into the reinforcement learning process, as it significantly improves the agent’s ability to learn and perform tasks in complex environments.

**Fig 6:**
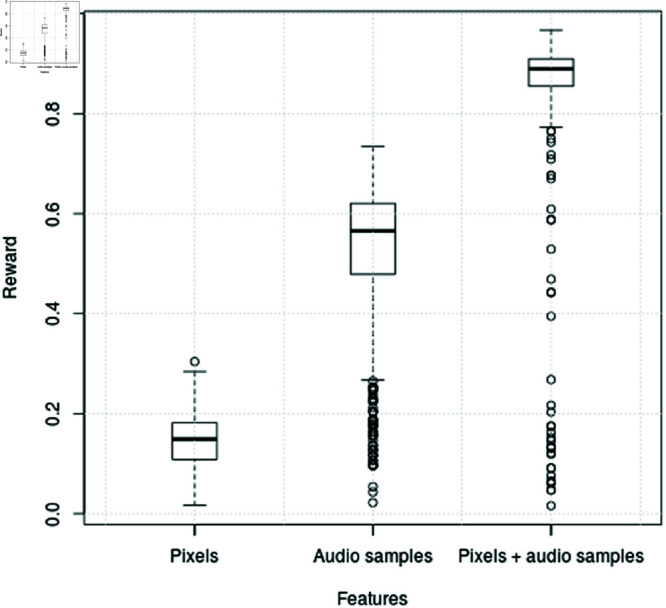
Box plot of Unity ML experiments using visual information, audio samples and combination of visual information and audio samples.

Fig 6 offers a comprehensive view of the reward ranges associated with different feature sets, namely visual information alone, audio samples alone, and visual information combined with audio samples, in the Unity ML environment. The box plot visualizes key statistics such as the minimum, lower quartile, median, upper quartile, and maximum reward for each feature set.

Notably, the plot highlights distinct patterns in reward distribution across the different feature combinations. Specifically, the system utilizing visual information together with audio samples exhibits the highest mean and median rewards, suggesting superior performance compared to other configurations. Conversely, relying solely on visual information yields the lowest mean and median rewards.

Furthermore, the plot showcases the variability in reward outcomes, as evidenced by the differences in minimum and maximum values across the feature sets. Interestingly, the combination of visual information and raw audio samples demonstrates the highest minimum and maximum reward values, indicating the potential for both improved performance and occasional exceptional outcomes when leveraging multimodal sensory inputs.

Overall, the box plot provides valuable insights into the distribution and variability of rewards associated with different feature combinations, underscoring the effectiveness of incorporating raw audio samples alongside visual information for enhancing the agent’s performance in the Unity ML environment.

Introducing occasional raw audio samples alongside visual information presents an interesting approach to leveraging multimodal sensory inputs during training. This experiment aims to assess the impact of intermittently incorporating audio cues on the agent’s learning and decision-making process, particularly in scenarios where visual information alone may be sufficient.

By varying the probability of receiving raw audio samples at each time step, ranging from 25% to 75%, you can examine how different levels of audio input affect the agent’s performance compared to the baseline system that relies solely on visual information. This analysis allows for a nuanced understanding of when and to what extent raw audio samples contribute to the agent’s decision-making process and overall task performance.

The results of this experiment can provide insights into the optimal balance between visual and auditory cues in reinforcement learning tasks, shedding light on the relevance and utility of raw audio samples in different environmental contexts. Moreover, it offers practical implications for designing more efficient and adaptive multimodal reinforcement learning systems tailored to specific task requirements and environmental conditions.

**Fig 7:**
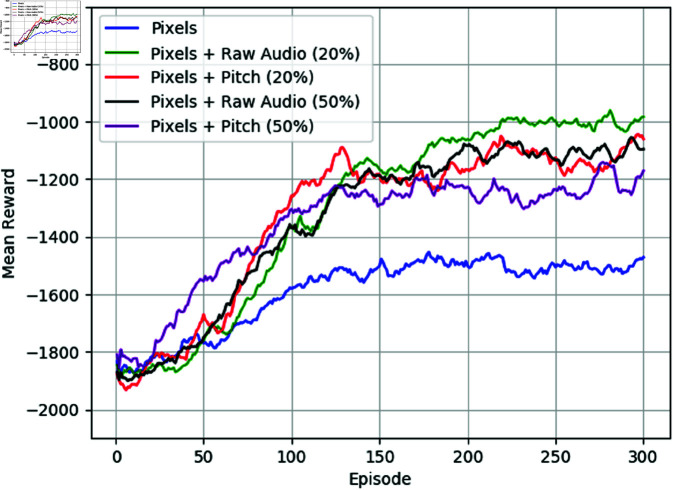
Mean reward of using only pixels and pixels with raw audio samples in ViZDoom environment. The audio features are occasionally used with 20% and 50% probability chances.

**Fig 8:**
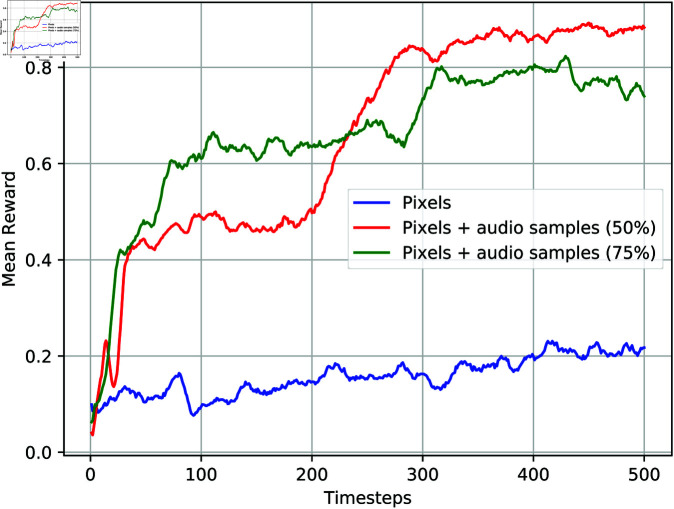
Mean reward of using visual information, visual information with raw audio samples in Unity ML environment. The raw audio samples are occasionally used with 50% and 75% probability chances.

Fig 7 and Figure 8 provide additional insights into the performance of the reinforcement learning agents when raw audio samples are occasionally introduced alongside visual information in ViZDoom and Unity ML environments, respectively. These figures complement the findings from experiments where raw audio samples are consistently used throughout training.

The learning curves depicted in these figures suggest that intermittently providing raw audio samples yield improvements in the average reward compared to using only visual information. This indicates that even occasional auditory cues can enhance the agent’s ability to navigate and accomplish tasks in complex environments. Moreover, similar to the results observed when raw audio samples are consistently used, the occasional introduction of audio features facilitates successful target acquisition by the learning agent.

These findings underscore the value of multimodal sensory inputs in reinforcement learning, highlighting the potential benefits of integrating auditory cues alongside visual information to enhance the agent’s decision-making process and overall performance. Furthermore, this approach offers flexibility in adapting to diverse environmental conditions, allowing the agent to leverage relevant sensory modalities as needed to effectively accomplish tasks.

The results summarized in Table 2 provide further insights into the performance of reinforcement learning agents trained with visual information and raw audio samples in both ViZDoom and Unity environments. The average rewards obtained over the last 100 episodes serve as a metric for evaluating the effectiveness of different input modalities.

**Table 2:** Mean scores of the last 100 episodes using PPO and DQN in Unity environment and ViZDoom.

gray Reinforcement Learning System	PPO with curiosity	PPO without curiosity	DQN
visual information	0.18	18	-1645
visual information + raw audio samples (25%)	0.84	85	-648
visual information + raw audio samples (50%)	0.86	87	-620
visual information + raw audio samples (75%)	0.85	89	-614
visual information + raw audio samples (100%)	0.89	0.91	-621

Consistently, the table highlights that utilizing raw audio samples alongside visual information leads to higher average rewards compared to using visual information alone. Moreover, the highest average rewards are achieved when raw audio samples are consistently incorporated into the agent’s input data. This trend underscores the importance of multimodal sensory inputs, particularly in scenarios where visual cues alone may not suffice for effective decision-making and task completion.

Furthermore, the variability in rewards across different experimental conditions demonstrates the robustness and generalizability of the proposed approach. By leveraging both visual and auditory information, reinforcement learning agents can adapt to diverse environmental conditions and achieve superior performance in challenging tasks.

The experimental results presented here validate the efficacy of raw audio samples for reinforcement learning tasks in both ViZDoom and Unity ML environments. These findings underscore the potential of multimodal learning approaches and highlight the importance of incorporating auditory cues to enhance the capabilities of learning agents, particularly in complex and dynamic environments where visual information alone may be insufficient.

## 6 Conclusions

This study highlights a critical gap in current reinforcement learning methodologies, which predominantly rely solely on visual information to interpret environments and make decisions. Yet, humans leverage various sensory inputs, including auditory cues, for enhanced perception and action. Notably, individuals with visual impairments face significant challenges in tasks such as playing video games solely relying on visual data. Therefore, integrating audio inputs becomes pivotal in such contexts. Consequently, we propose the utilization of raw audio samples alongside visual information for reinforcement learning tasks, assessing their efficacy across Unity and ViZDoom environments using DQN and PPO algorithms.

Our experimental findings across ViZDoom and Unity ML environments underscore the benefits of supplementing visual data with complementary audio samples. Specifically, incorporating audio features alongside visual cues leads to improved average rewards compared to relying solely on visual inputs. Moreover, our results highlight the utility of audio features in scenarios where visual information may be limited or absent.

The empirical outcomes of our study underscore the significance of raw audio samples in reinforcement learning, emphasizing their role in enhancing agent performance. Future investigations could extend these findings to diverse reinforcement learning environments and tasks, such as alternative video games or high-fidelity audio simulations. Additionally, exploring the specific auditory cues the agent learns to leverage for decision-making warrants further analysis. Another intriguing avenue involves comparing agents utilizing recurrent neural network architectures, such as Long-Short Term Memory, with and without access to audio information, to elucidate the impact of audio inputs on learning dynamics.

## References

[1] Ernst D, Louette A. Introduction to reinforcement learning. In: Feuerriegel S, Hartmann J, Janiesch C, Zschech P. Generative AI. Bus Inf Syst Eng. 2024;66:111–26.

[2] Sutton R, Barto A, Bach F. Introduction to reinforcement learning. Cambridge: MIT Press; 1998.

[3] Kaelbling LP, Littman ML, Moore AW. Reinforcement learning: a survey. J Artif Intell Res. 1996;4:237–85. doi: 10.1613/jair.301

[4] Bellman R. A Markovian decision process. Indiana Univ Math J. 1957;6(4):679–84. doi: 10.1512/iumj.1957.6.56038

[5] Solomon E, Cios KJ. FASS: face anti-spoofing system using image quality features and deep learning. Electronics. 2023;12(10):2199. doi: 10.3390/electronics12102199

[6] Heess N, Wayne G, Silver D, Lillicrap T, Erez T, Tassa Y. Learning continuous control policies by stochastic value gradients. In: Advances in Neural Information Processing Systems. 2015, pp. 2944–52.

[7] Mnih V, Kavukcuoglu K, Silver D, Rusu AA, Veness J, Bellemare MG, et al. Human-level control through deep reinforcement learning. Nature. 2015;518(7540):529–33. doi: 10.1038/nature14236 25719670

[8] Levine S, Finn C, Darrell T, Abbeel P. End-to-end training of deep visuomotor policies. J Mach Learn Res. 2016;17:1334–73.

[9] Shalev-Shwartz S, Shammah S, Shashua A. Safe, multi-agent, reinforcement learning for autonomous driving. arXiv, preprint. 2016.

[10] Juliani A, Berges V, Vckay E, Gao Y, Henry H, Mattar M, et al. Unity: a general platform for intelligent agents. arXiv, preprint. 2018.

[11] Solomon E, Cios K. HDLHC: hybrid face anti-spoofing method concatenating deep learning and hand-crafted features. In: 2023 IEEE 6th International Conference on Electronic Information and Communication Technology (ICEICT). 2023, pp. 470–4.

[12] Sallans B, Hinton G. Reinforcement learning with factored states and actions. J Mach Learn Res. 2004;5:1063–88.

[13] Solomon E. Face anti-spoofing and deep learning based unsupervised image recognition systems. Scholarscompass.vcu.edu. 2023.

[14] Solomon E, Woubie A, Cios KJ. UFace: an unsupervised deep learning face verification system. Electronics. 2022;11(23):3909. doi: 10.3390/electronics11233909

[15] Solomon E, Woubie A, Emiru ES. Self-supervised deep learning based end-to-end face verification method using Siamese network. In: 2023 IEEE International Conference on Service Operations and Logistics, and Informatics (SOLI). IEEE; 2023, pp. 1–6. doi: 10.1109/soli60636.2023.10425759

[16] Solomon E, Woubie A, Emiru ES. Nearest neighbor based unsupervised deep learning image recognition method. In: 2023 International Conference on Modeling, Simulation &amp; Intelligent Computing (MoSICom). IEEE; 2023, pp. 592–6. doi: 10.1109/mosicom59118.2023.10458764

[17] Solomon E, Woubie A. Federated learning method for preserving privacy in face recognition system. arXiv, preprint. 2024.

[18] Graves A, Mohamed A, Hinton G. Speech recognition with deep recurrent neural networks. In: 2013 IEEE International Conference on Acoustics, Speech and Signal Processing. IEEE; 2013, pp. 6645–9. doi: 10.1109/icassp.2013.6638947

[19] Russakovsky O, Deng J, Su H, Krause J, Satheesh S, Ma S, et al. ImageNet large scale visual recognition challenge. Int J Comput Vis. 2015;115(3):211–52. doi: 10.1007/s11263-015-0816-y

[20] Lederle M, Wilhelm B. Combining high-level features of raw audio waves and mel-spectrograms for audio tagging. arXiv, preprint, 2018.

[21] Piczak KJ. Environmental sound classification with convolutional neural networks. In: 2015 IEEE 25th International Workshop on Machine Learning for Signal Processing (MLSP). IEEE; 2015, pp. 1–6. doi: 10.1109/mlsp.2015.7324337

[22] Vinyals O, Ewalds T, Bartunov S, Georgiev P, Vezhnevets A, Yeo M, et al. Starcraft II: a new challenge for reinforcement learning. arXiv, preprint, 2017.

[23] Zhang J, Fu X. The influence of background music of video games on immersion. J Psychol Psychother. 2015;5(1).

[24] Zénouda H. New musical organology: the audio-games. In: MISSI’12 - International Conference on Multimedia & Network Information Systems, 2012.

[25] Yuan B. Towards generalized accessibility of video games for the visually impaired. Reno: University of Nevada; 2009.

[26] Qian X, Zhong Z, Zhou J. Multimodal machine translation with reinforcement learning. arXiv, preprint, 2018.

[27] Jiang A-W, Liu B, Wang M-W. Deep multimodal reinforcement network with contextually guided recurrent attention for image question answering. J Comput Sci Technol. 2017;32(4):738–48. doi: 10.1007/s11390-017-1755-6

[28] Zhang J, Zhao T, Yu Z. Multimodal hierarchical reinforcement learning policy for task-oriented visual dialog. arXiv, preprint, 2018.

[29] Muslea I, Minton S, Knoblock CA. Active learning with multiple views. J Artif Intell Res. 2006;27:203–33. doi: 10.1613/jair.2005

[30] Kempka M, Wydmuch M, Runc G, Toczek J, Jaśkowski W. ViZDoom: A doom-based AI research platform for visual reinforcement learning. In: 2016 IEEE Conference on Computational Intelligence and Games (CIG). 2016, pp. 341–8.

[31] Solomon E, Woubie A, Emiru E. Unsupervised deep learning image verification method. arXiv, preprint, 2023.

[32] Solomon E, Woubie A, Emiru E. Autoencoder based face verification system. arXiv, preprint, 2023.

[33] Schulman J, Wolski F, Dhariwal P, Radford A, Klimov O. Proximal policy optimization algorithms. arXiv, preprint, 2017.

[34] OpenAI. OpenAI five. 2018.

[35] Woubie A, Solomon E, Emiru E. Image clustering using restricted Boltzmann machine. arXiv, preprint, 2023.

[36] Hill A, Raffin A, Ernestus M, Gleave A, Kanervisto A, Traore R, et al. Stable baselines. Available from: https://github.com/hill-a/stable-baselines. 2018.

[37] Lee L. Robotic search and rescue via online multi-task reinforcement learning. arXiv, preprint, 2015.

[38] Software doom. New York: GT Interactive; 1993.

[39] Watkins CJ, Dayan P. Technical note: Q-learning. Mach Learn. 1992;8:279–92. doi: 10.1023/A:1022676722315

[40] Pathak D, Agrawal P, Efros A, Darrell T. Curiosity-driven exploration by self-supervised prediction. In: Proceedings of the IEEE Conference on Computer Vision and Pattern Recognition Workshops. 2017, pp. 16–7.

[41] Henderson P, Islam R, Bachman P, Pineau J, Precup D, Meger D. Deep reinforcement learning that matters. In: Proceedings of the Thirty-Second AAAI Conference on Artificial Intelligence. 2018.

[42] Solomon E, Woubie A, Emiru E. Deep learning based face recognition method using Siamese network. arXiv, preprint, 2023.

[43] Zeiler M. Adadelta: an adaptive learning rate method. arXiv, preprint, 2012.

